# Low Density Lipid Nanoparticles for Solid Tumor Targeting

**DOI:** 10.3797/scipharm.1401-10

**Published:** 2014-08-28

**Authors:** Mayank Shrivastava, Aviral Jain, Arvind Gulbake, Pooja Hurkat, Neeti Jain, R. Vijayraghwan, Sanjay K. Jain

**Affiliations:** ^1^ Pharmaceutics Research Project Laboratory, Department of Pharmaceutical Sciences, Dr. H. S. Gour University, Sagar (M. P.), India.; ^2^ Defense Research and Development Establishment, Gwalior (M. P), India.

**Keywords:** LDL receptors, Lipoproteins, Apo protein 100, 5-Fluorouracil

## Abstract

One of the most significant characteristics of cancer cells is their rapid dividing ability and overexpression of LDL receptors, which offers an opportunity for the selective targeting of these cells. 5-Fluorouracil (5-FU)-encapsulated low density lipid nanoparticles (LDLN) were prepared by the emulsion congealing method which mimics the plasma-derived LDL by acquiring the apolipoprotein B-100 from the blood. The average particle size, transmission electron microscope (TEM), and drug content of the prepared LDLN dispersion were found to be 161±3.5 nm, with spherical shape, and 0.370±0.05 mg/mL, respectively. *In vitro* release studies revealed a sustained profile which decreased with a lapse of time. *In vivo* studies of 5-FU serum concentration and biodistribution revealed a 5-FU serum concentration of 8.5% in tumor cells and about 2.1% in the liver at the end of 24 hr from LDLN. Tumor growth suppression studies showed 185.42% average tumor growth and 89.76% tumor height as compared to the control exhibiting tumor growth at 1166.47% and tumor height at 176.07%. On the basis of these collective data, it is suggested that a higher accumulation of LDLN, when given as an IV, in solid tumors is attributed to the active uptake of LDLN via LDL receptors via apolipoprotein B-100.

## Introduction

Surgery is the most widely accepted approach for the treatment of solid tumors because of its effectiveness, whereas chemotherapeutic agents and radiotherapeutics are non-selective for cancer cells. However, incomplete removal during surgery makes cancer an unsolved problem. This gives rise to the need of developing a carrier system for selective targeting and complete removal of cancer cells.

Generally, most of the body’s nutrients are transported through the blood i.e. circulatory fluid which is hydrophilic in nature. On the other hand, for the transport of unbound lipophilic substances**,** it utilizes some amphiphilic systems or molecules such as lipoproteins which serve as natural biological carriers and transport various types of lipids in the blood circulation.

It is reported by many scientists that cytotoxic drugs could be incorporated into lipoproteins without changing the integrity of the native lipoprotein structure [1–6]. Lipoproteins as drug carriers offer several advantages which include 1) their endogenous nature and hence, do not trigger immunological responses; 2) they have a long circulating half-life; 3) they have a particle size in the nanometer range, allowing the diffusion from the vascular to the extravascular compartments; 4) lipoproteins can potentially serve as the carriers for targeted drug delivery through specific cellular receptors e.g., low density lipoprotein (LDL) drug complexes used to target cancer cells having higher LDL receptor expression than normal cells; 5) the lipid core of the lipoprotein provides a suitable compartment for carrying drugs [7, 8].

If we look at the structural constitution of LDL, it is composed of a core of hydrophobic lipids, primarily cholesteryl esters, with a small amount of triglyceride, and has a surface coat of phospholipids, unesterified cholesterol, and a single molecule of apolipoprotein B-100 (apoB) [9]. ApoB-100 is a 550,000 D glycoprotein with nine amino acids (3359-3367) serving as the binding domain for the LDL receptor [10]. Cholesterol is transported by LDL via LDL receptors on the cell’s surface and used for cell growth and membrane repair.

It is reported that these LDL receptors are overexpressed on different types of solid tumors like colon cancer [11], prostate tumors [12], adrenal tumors [13], hormone unresponsive breast tumors [14], cancers of gynecological origin [15], malignant brain tumors [16], lung tumor tissues [17], and leukemia [18–21]. The reason might be that in a pathological condition like solid tumor growth, the rapidly dividing cells require high cholesterol content and therefore, LDL receptors are overexpressed so that plasma-derived LDL could mobilize the cholesterol for the rapidly dividing cancer cells. It was also suggested that plasma-derived LDL could be used as a drug delivery system for tumors expressing LDLR (low density lipid receptors) since its hydrophobic core has the possibility of incorporating lipophilic drugs [22, 23]. Drugs have been either directly loaded onto plasma LDL or the core lipids of LDL were replaced with drugs [24–30]. However, it is very tedious and expensive to use the plasma-derived LDL as a drug carrier because of its separation, drug loading, and administration which requires sophistication. An alternative approach used reconstituted LDL consisting of a lipid emulsion containing a drug stabilized by purified ApoB-100 [31–34]. The ApoB-100 protein is difficult to isolate due to its large size and propensity to aggregate and is therefore not useful for the generation of large batches of reconstituted LDL.

**Fig. 1.**
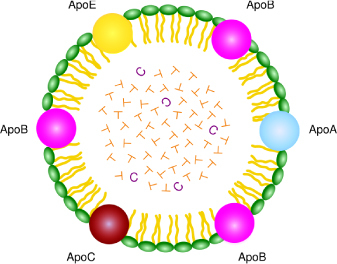
A typical nano-LDL

Studies have shown the feasibility of creating a synthetic LDL particle as a replacement for serum LDL in cell culture media by using a lipid emulsion and a peptide composed of the LDLR binding domain of ApoB [35, 36]. These synthetic particles were able to support cell growth by delivering cholesterol to cells via the LDL receptor. Current hypotheses utilize the synthetic nano-LDL (nLDL) particles which mimic the binding and uptake properties of plasma-derived LDL for targeting the solid tumor (Fig. 1).

## Materials and Methods

### Materials

Cholesteryloleate, triolein, egg lecithin, and cholesterol were purchased from Sigma Chemicals, (St. Louis, MO, USA). 5-Fluorouracil was obtained as a gift sample from Shalak Pharamaceutical Pvt. Ltd. New Delhi, India. All other chemicals used were of analytical reagent grade and were used as received. HPLC grade water was used.

### Preparation of Low Density Lipid Nanoparticles (LDLN)

LDLN were prepared by the emulsion congealing method [37]. Lipids (cholesteryloleate, triolein, egg lecithin) were dissolved in a mixture of chloroform and methanol (2:1 v/v). Then solvents were evaporated under the flow of nitrogen to get solid lipids. The drug (5-fluorouracil, 5-FU) was dissolved in methanol separately and added to the solid lipids and mixed thoroughly followed by the evaporation of methanol under the flow of nitrogen to get the drug-incorporated solid lipids. Tris buffer (20 μM) pH 7.2 was added to the drug-incorporated solid lipids with continuous vortexing for 15 min and sonicated for 3 min at 0–4°C using a probe sonicator (Lark Innovative Tecknowledge, India), to obtain a dispersion of low density lipid nanoparticles. The unentrapped drug was removed by the mini column centrifuge method [38]. These LDLN were further characterized for shape morphology, particle size, zeta potential, *in vitro* drug release, and drug content.

### Preparation of Solid Lipid Nanoparticles

Solid lipid nanoparticles (SLN) were prepared using the similar method as reported above for the preparation of LDLN [37], utilizing lipids such as egg lecithin, tristearin, and cholesterol. These SLN were characterized for their shape morphology, particle size, zeta potential, *in vitro* drug release, and drug content.

### Shape Morphology

Formulations of LDLN and SLN were characterized for their shape using transmission electron microscopy (TEM). All samples were examined under a transmission electron microscope (Philips Morgagni 268, Eindhoven, Netherlands) at an acceleration voltage of 100 kV, and photomicrographs were taken at a suitable magnification.

### Particle Size and Zeta Potential

The average particle size and zeta potential of LDLN and SLN were determined by the photon correlation spectroscopy-based Zetasizer (Zetasizer 3000, Malvern Instruments, Malvern, UK). The dispersions were diluted 1:9 v/v with deionized water. Triplicate measurements were made for each sample.

### In Vitro Drug Release

*In vitro* drug release from 5-FU-entrapped LDLN and SLN were studied using a dialysis bag membrane method. The dispersions of LDLN and SLN free from the unentrapped drug (2 ml, ~10 mg) were taken into a dialysis bag (molecular weight cutoff 10,000 Da, Hi Media India). The bag was suspended in a beaker containing 100 ml of a saline phosphate buffer (pH 7.4) under continuous stirring using a hot plate magnetic stirrer, maintained at 37±1°C. Samples were withdrawn periodically and replaced with the equivalent volume of the fresh saline phosphate buffer (pH 7.4) and the amount of drug was quantified using an HPLC method [39].

### Drug Content

Drug content was determined after removing the unentrapped drug using the mini column centrifuge method [38]. The amount of entrapped drug in LDLN and SLN was determined by disrupting the particles using triton-X 100 (1% v/v) and the drug concentration was quantified by an HPLC method [39].

### In Vivo Studies

*In vivo* studies were performed with the permission of the Animal Ethical Committee, Dr. H. S. Gour University, Sagar (M.P.).

### Tumor Induction in Animals

*In vivo* studies were performed on female Swiss mice (weighing 20–30 g) (n=3). Ehlrich’s ascites carcinoma (EAC) cell lines were cultured in the peritoneal cavity of Swiss mice. After sufficient growth, cells were taken and suspended in saline phosphate buffer (pH 7.4) and centrifuged at 2000 rpm for 10 min. Supernatant was discarded and cells were resuspended in PBS buffer (pH 7.4) and counted in Neubaure’s chamber using a microscope (LEICA, DMIRB). Further dilutions were prepared to get 5 × 10^6^ viable cells per 0.2 mL of the suspension and injected subcutaneously in the back of mice for generation of a solid tumor. After 20 days, animals with the desired tumor size were selected for further studies.

### In Vivo Biodistribution

Tumor-bearing female Swiss mice (weighing 20–30 g) were taken and divided into four groups with 18 mice in each group. They were fasted overnight before administration of the developed formulations. The first group was intravenously administered with an aqueous solution of 5-FU (dose of 3 mg/kg body weight). Animals of the second and third group were administered (IV) with 5-FU-entrapped LDLN and SLN (5-FU equivalent to about 3 mg/kg body weight), respectively. The fourth group served as a control.

One animal from each group was sequentially sacrificed after 15 min, 30 min, 1 hr, 2 hr, 4 hr, and 24 hr after administration and the blood was collected by cardiac puncture. The tumor and different organs i.e. heart, liver, spleen, lungs, and kidney were excised, isolated, dried with tissue paper, and weighed. The amount of drug present in each organ as well as the serum concentration was determined using an HPLC method [39]. All the experiments were performed in triplicate.

### Tumor Growth Suppression Studies

To observe the effect of the developed formulations on the tumor growth, 24 tumor-bearing mice were selected with measurable tumor size and divided into four groups. Animals of the first group which served as controls were subjected to administration (IV) of 0.2 ml of normal saline solution for 15 days. Animals of second, third, and fourth group were administered (IV) drug-entrapped LDLN and SLN, respectively. The dose of the drug in each case was given according to 3 mg/kg body weight. Simultaneously, atorvastatin solution was applied topically (1 mg/kg body weight per day) on the tumor to increase the expression of the receptors**.** The measurement of tumor growth was done and expressed as the percentage growth in tumor volume and tumor height. The tumor volume was calculated using the formula used for calculating the volume of ellipsoid structure [40].

V = (π/6) a. b^2^

Where,

a = longest diameter of the tumor {length}

b = diameter perpendicular to a {width}

All the experiments were performed in triplicate.

### Statistical Analysis

Data analysis was carried out using the software package Microsoft Excel®, Version 2000. Statistically significant differences were determined using the analysis of variance (ANOVA) with *P* <0.05 as a minimal level of significance.

## Results

5-FU-entrapped LDLN and SLN were prepared using the emulsion congealing technique. Low density lipid nanoparticles (LDLN) and solid lipid nanoparticles (SLN) were characterized for shape morphology, particle size, and *in vitro* drug release and drug content.

**Fig. 2.**
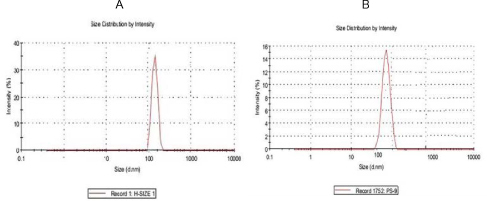
Size distribution curve for A) LDLN and B) SLN

**Fig. 3.**
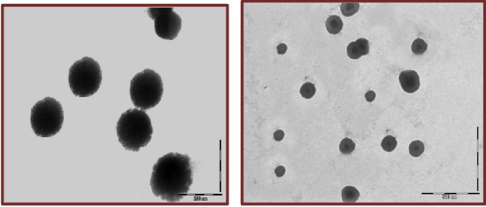
Transmission electron microscope photomicrograph of (A) LDLN and (B) SLN

The average particle size of the prepared LDLN and SLN was found to be 161±3.5 nm and 158±2.7 nm, respectively (Figure 2 A and B). The transmission electron microscope (TEM) photomicrograph (Fig. 3) showed that LDLN and SLN were spherical in shape and do not show considerable variation in the shape. Drug contents for the dispersions of LDLN and SLN were found to be 0.370±0.05 mg/ml and 0.374±0.045 mg/mL, respectively.

**Fig. 4.**
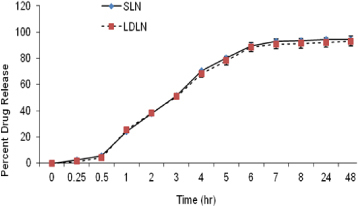
*In vitro* percentage drug release for SLN and LDLN (n=3), p≤0.05

*In vitro* release studies of LDLN revealed the sustained release of the drug which decreased with the lapse of time up to 93.09% drug release at the end of 48 hr (Fig. 4). While the *in vitro* drug release profile of SLN shows a similar behavior to LDLN, i.e., about 94.2% of the drug was found to be released in 48 hr (Fig. 4). SLNs and LDLNs both showed biphasic release patterns, with the initial burst release of about 1.5 to 2.5 percent attributed to the aqueous solubility of 5-FU leading to a rapid dissolution of the drug molecules present in the surface layer of the particles. After the 6^th^ hr, the release became slow, leading to a sustained release pattern.

**Fig. 5.**
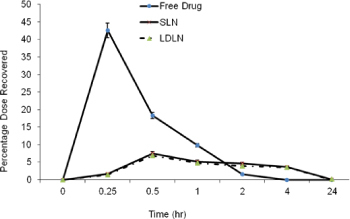
Drug serum profile of the drug solution, SLN and LDLN (n=3)

The *in vivo* biodistribution study results show that the serum concentration of the plain drug was found to be 42.6% in 15 min, while only 1.5% and 1.7% of the drug was recovered from LDLN and SLN, respectively. Further, the plain drug was rapidly cleared from the blood after 2 hr while the drug recovered from LDLN was about 3.5% and that from SLN was about 4.6% after 2 hr (Fig. 5).

**Fig. 6.**
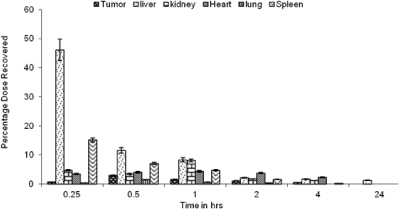
Organ distribution profile of 5-FU after IV administration of the drug solution (n=3), p≤0.05

The organ distribution pattern of the plain drug showed 46.1% in the liver, 15.2% in the spleen, and 0.7% in the tumor within the first 15 min of administration (Fig. 6).

**Fig. 7.**
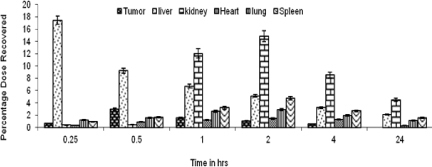
Organ distribution profile of 5-FU after IV administration of SLN (n=3)

**Fig. 8.**
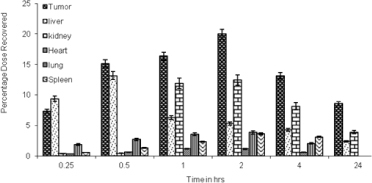
Organ distribution profile of 5-FU after IV administration of LDLN (n=3)

After 2 hr, the drug distribution in the tumor cells was increased to about 1.9%, while in the liver and the spleen it was reduced. Drug distribution from LDLN and SLN within 15 min was found to be 7.3% and 1.8%, respectively, in the tumor cells which increased up to 19.9% for LDLN and 6.1% for SLN after 2 hr. Drug distribution at the end of 24 hr for LDLN was about 8.5% and 5.8% for SLN (Fig. 7 and 8).

Whereas the organ distribution of LDLN and SLN showed about 17.5% and 9.3% of the drug recovered from the liver, 0.9% and 0.5% was recovered from the spleen, respectively, in the initial 15 min. At the end of 2 hr, the drug recovered was about 5.1% and 5.4% from the liver and 3.6% and 4.7% from the spleen for LDLN and SLN, respectively. At the end of 24 hr, about 2.1% and 2.4% of the drug was recovered from the liver for LDLN and SLN, respectively. While the drug recovered from the spleen was about 1.5% for SLN, no appreciable amount was recovered for LDLN (Fig. 7 and 8).

**Fig. 9.**
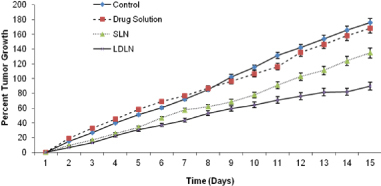
Comparison between the average percentage tumor growth profiles of the groups (n=3)

Tumor growth suppression studies were performed on tumor-bearing Swiss mice. Animals of the first group (control) exhibited the highest average percentage tumor growth of 1166.47%, whereas the animals of the second group (plain drug) showed 692.10% average percent growth. Animals of the third group administered with LDLN exhibited an average of 185.42% growth (Fig. 9).

**Fig. 10.**
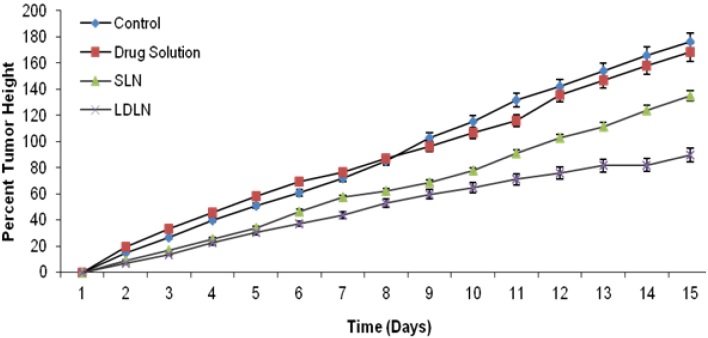
Comparison between the average percentage growth in the tumor height of groups (n=3), p ≤ 0.05

The animals administered with SLN i.e., the fourth group showed an average of 426.58% growth. This is approximately a threefold difference when compared with the control animals. When tumor height was measured, the control group (first group) and second group (plain 5-FU) revealed tumor heights of 176.07% and 168.26%, respectively, whereas LDLN and SLN reduced the tumor height comparatively i.e., 89.76% and 134.81% in the case of LDLN and SLN, respectively (Fig. 10).

## Discussion

Surgery is the most widely accepted approach for the treatment of solid tumors because of its effectiveness, whereas chemotherapeutic agents and radiotherapeutics are non-selective for cancer cells. However, incomplete removal during surgery makes cancer an unsolved problem. This gives rise to the need of developing a carrier system for selective targeting and complete removal of cancer cells**.**

In cancerous pathology, cells overexpress LDL receptors and hence, offer an opportunity for selective targeting of these cells. Plasma-derived LDL could be used as a drug carrier and artificial lipoproteins of similar composition can be used with the receptor binding domain of the apolipoprotein B-100 as the ligand, but both of the processes are complicated and expensive. Hence, to solve this problem, low density lipid nanoparticles (LDLN) were prepared from a lipid emulsion which mimics the plasma-derived LDL by acquiring the apolipoprotein B-100 from the blood [41].

Preparation of 5-FU-entrapped LDLN and SLN using the emulsion congealing technique offers clear advantages over the existing methods, such as the complete removal of pharmaceutically unacceptable organic solvents, no high-pressure homogenization, easy handling, and a fast production process [42]. Low density lipid nanoparticles (LDLN) and solid lipid nanoparticles (SLN) were compared and characterized on various parameters. The average particle size of the developed LDLN and SLN were more and less similar, respectively, with spherical shape as revealed by the TEM images. Moreover, the drug content value also suggests that their equivalent drug entrapping ability might be due to the variation in their constitution of lipids and the mechanism of uptake in the body. However, this does not affect the *in vitro* drug release pattern from both formulations, which mimic a sustained release profile.

*In vitro* drug release studies revealed a similar release profile for both formulations, which would be due to the use of more or less similar lipidic constitutions in both of the formulations and hence, approximately a similar drug release pattern.

The serum concentration of the plain drug was more than 40 percent as compared to that from LDLN and SLN within 15 min. In the initial 15 min, the LDLN and SLN exhibited a similar serum recovery of the drug, however with a lapse of time, i.e., after 2 hr, the LDLN sustained drug release in the serum due to the influence of the ligand anchoring to the surface of LDLN, as compared to SLN, which was not sustained. While in the case of the plain drug, it was rapidly cleared from the blood after 2 hr because of the short half-life.

The organ distribution pattern showed that the administration of the plain drug led to the maximum accumulation in the liver and a less effective concentration reaching tumor cells even after 2 hr of administration. However, on comparing the total drug distribution profile in the tumor cells, a good concentration of the drug was found, in case of LDLN. This may be attributed due to the active targeting mechanism, which involves LDL receptors present on the tumor cells, thus aiding in the preferential uptake of LDLN, while SLN are taken up by a passive uptake mechanism. The uptake of LDLN mimics the plasma-derived LDL. It also leads us to the fact that LDLN and SLN have the additional advantage of lessening hepatotoxicity as compared to the plain drug when administered alone and thus increasing drug bioavailability at the targeted tumor site.

In the tumor growth suppression studies, it was observed that the animals of the first group (control) exhibited the highest average percentage tumor growth of 1166.47%, due to the lack of antineoplastic drugs, while the animals of the second group (plain 5-FU) showed 692.10% average percent growth due to the less effective uptake of plain 5-FU because of less vasculature or blood supply in the solid tumor mass leading to less bioavailability and therapeutic response. Among the animals of the third and fourth groups administered with LDLN and SLN, the third group of animals showed a lower average percent growth rate than the fourth group i.e., 185.42% < 426.58% growth because the active uptake of LDLN via LDL receptors increased the concentration of 5-FU inside the solid tumor cells. This is approximately a threefold difference when compared with the control animals. In the case of tumor height, it was observed that the LDLN-administered group (group three) showed 89.76% of height which is also attributed to the active uptake of LDLN in solid tumor cells.

## Conclusion

On the basis of the obtained results and by keeping the physiological mechanisms of the body in mind, we can hypothesize that the reason behind higher accumulation of LDLN in tumors is due to “active targeting”. When the formulation is given as an IV, beta-apoprotein of the blood becomes attached to the surface of the nanoparticles (LDLN) and function as a ligand, thus imparting the affinity to the carrier to be actively taken up by tumor cells which overexpress the LDL receptors during their growth. By this process, the nanoparticles (LDLN) are taken up by the tumor cells via LDL receptor. In this way, the developed formulation has a great potential for active tumor targeting. This mechanism can also be utilized in targeting the solid tumors with the help of statins in the liver and adrenal glands, which also overexpress the LDL receptors. The present work also leads to the conclusion that such a particulate system exhibits higher drug loading, selective drug targeting, and higher bioavailability when injected.
